# An investigation of factors associated with the health and well-being of HIV-infected or HIV-affected older people in rural South Africa

**DOI:** 10.1186/1471-2458-12-259

**Published:** 2012-04-02

**Authors:** Makandwe Nyirenda, Somnath Chatterji, Jane Falkingham, Portia Mutevedzi, Victoria Hosegood, Maria Evandrou, Paul Kowal, Marie-Louise Newell

**Affiliations:** 1 Africa Centre for Health and Population Studies, University of KwaZulu-Natal, PO Box 198 , R618 Enroute Somkhele, Mtubatuba, 3935, South Africa; 2 School of Social Sciences, University of Southampton, Highfield, Southampton, UK; 3 Multi-Country Studies Unit, World Health Organization, Geneva, Switzerland; 4 Centre for Paediatric Epidemiology and Biostatistics, UCL Institute of Child Health, London, UK

**Keywords:** South Africa, Older people, Health status, Functional ability, Quality of life

## Abstract

**Background:**

Despite the severe impact of HIV in sub-Saharan Africa, the health of older people aged 50+ is often overlooked owing to the dearth of data on the direct and indirect effects of HIV on older people’s health status and well-being. The aim of this study was to examine correlates of health and well-being of HIV-infected older people relative to HIV-affected people in rural South Africa, defined as participants with an HIV-infected or death of an adult child due to HIV-related cause.

**Methods:**

Data were collected within the Africa Centre surveillance area using instruments adapted from the World Health Organization (WHO) Study on global AGEing and adult health (SAGE). A stratified random sample of 422 people aged 50+ participated. We compared the health correlates of HIV-infected to HIV-affected participants using ordered logistic regressions. Health status was measured using three instruments: disability index, quality of life and composite health score.

**Results:**

Median age of the sample was 60 years (range 50–94). Women HIV-infected (aOR 0.15, 95% confidence interval (CI) 0.08–0.29) and HIV-affected (aOR 0.20, 95% CI 0.08–0.50), were significantly less likely than men to be in good functional ability. Women’s adjusted odds of being in good overall health state were similarly lower than men’s; while income and household wealth status were stronger correlates of quality of life. HIV-infected participants reported better functional ability, quality of life and overall health state than HIV-affected participants.

**Discussion and conclusions:**

The enhanced healthcare received as part of anti-retroviral treatment as well as the considerable resources devoted to HIV care appear to benefit the overall well-being of HIV-infected older people; whereas similar resources have not been devoted to the general health needs of HIV uninfected older people. Given increasing numbers of older people, policy and programme interventions are urgently needed to holistically meet the health and well-being needs of older people beyond the HIV-related care system.

## Background

South Africa is in the midst of a health transition characterised by four disease burdens: communicable, perinatal and maternal mortality, injury-related and non-communicable diseases [1-3]. The latter burden is a result of demographic transition largely characterised by declines in fertility [4,5] and improved survival at older ages, which has led to an increasing proportion of older people in South Africa [1,6]. This rapidly increasing proportion of older people is occurring in spite of the severe impact of HIV on adult mortality. It is projected that 15% of the total South African population in 2050 will be aged 60 years or over, up from around 8% of the total 2011 population [7]. This transition to an increasingly ageing society poses social, economic and health challenges.

The South African health care system is as yet not adequately prepared for and well-equipped to deal with the needs of older people and the associated rise in chronic conditions, nor is the health and well-being of older people in Africa well understood owing to the paucity of studies on the health status of older people [8-10], especially those from rural South Africa. In the few studies that have been conducted in South Africa, the HIV status of older people has not been explicitly studied [11-14], while others have focused solely on HIV-infected people of all ages [15-17]. Thus, in South Africa where HIV prevalence is a major health issue, there is limited reliable information on the physical, mental and social well-being of HIV-infected relative to HIV-affected older people.

In this paper we aimed to examine the correlates of health and well-being of HIV-infected older people aged 50 years and above, relative to their HIV-affected peers in rural South Africa. We defined HIV-affected older people as those with an HIV-infected adult child (18–49 years) or with an HIV-related death of an adult child between 2008 and 2010.

## Methods

### Study setting

Data used in this study were collected within the Africa Centre surveillance area, using instruments adapted from the World Health Organization (WHO) Study on global AGEing and adult health (SAGE) [18]. The Africa Centre surveillance area is situated in the Mpukunyoni tribal area, Hlabisa sub-district, northern KwaZulu-Natal. Since 2000, approximately 90,000 household members are monitored every year in 11,500 households; a third of whom are not currently resident in the surveillance area [19]. On 01 January 2010, there were 61 431 household members resident within the surveillance area, of whom 13% were aged 50 and above. The Africa Centre surveillance area is well geo-circumscribed and predominantly rural, albeit with a small urban segment (less than 10% of the surveillance population) around a local township. The population in our study area in rural South Africa is characterised by people living in predominantly multi-generational households consisting of grandparents, adults and children [20,21].

Demographic, social and health data on all members of the household are collected bi-annually from a household key informant. Data collected include births, deaths, population movements and household membership [19]. For each death recorded during the routine household visits, detailed cause of death information is collected by trained nurses within six months of the death being reported using a validated verbal autopsy data collection instrument [22]. These verbal autopsy forms are then passed on to two independent physicians who assign using the International Classification of Diseases version 10 (ICD-10) a cause of death [23]. In addition once a year, data are collected on socio-economic variables, such as household assets, access to electricity, sanitation facilities, government cash transfers, employment status, energy sources and educational attainment. Additionally data on sexual behaviour and HIV sero-status are collected annually from all adult household members (15 years and above) [19]. Details about the Africa Centre surveillance can be found elsewhere [19,20] or by visiting http://www.africacentre.com.

In many multi-generational households characteristic of our study area, people over 60 years of age in receipt of government old-age grants are the main source of income [24,25], given the high unemployment rate among adults [26,27]. In addition to the challenge of providing financial support to their households, given the high HIV burden in South Africa, older persons are also providing long-term personal and health care to their adult offspring infected with HIV and to younger children upon the death of their parents [28-34]. Furthermore, older people are at risk of becoming HIV-infected themselves [35-37], with additional numbers coming from HIV-infected adults on treatment living longer [38]. It is thus important to study the socio-economic and demographic factors influencing the health status of older people in rural South Africa.

### The SAGE well-being of older people study (WOPS)

The SAGE Well-Being of Older People Study (WOPS) was carried out from March-August 2010, using a shortened version of the SAGE instrument, and partially harmonized with a similar sub-study in Uganda [39]. The study instrument had three main components: 1) detailed questionnaire collecting basic demographic information and the health status of the older person, including functional ability assessment, subjective well-being, chronic health conditions and symptoms, health care utilisation, care-giving and -receiving, and the experience of living with HIV; 2) collection of anthropometric measurements; and finally, 3) blood samples providing laboratory measured health risk biomarkers for cardiovascular diseases, diabetes and hypertension. Data collected in the anthropometric measurements and the blood specimens were not used in describing the health and well-being of older people here as these were outside the scope of the present analysis.

The overall aim of WOPS was to investigate the direct and indirect effects of HIV on the health and wellbeing of people aged 50-plus years. The criteria for inclusion were being aged 50+ years, under observation and residing within the Africa Centre surveillance area. Other specific requirements were group-specific:

· group 1, a participant had to be HIV-infected and on treatment for one year or more;

· group 2, an individual had to be HIV-infected and on treatment for 3 months or less, or waiting to initiate antiretroviral treatment (ART);

· group 3 consisted of older people who had an adult child 18–49 years who was HIV-infected and either on treatment for one year or more, or for three months or less; and

· group 4 consisted of older people who had experienced death of an adult child between 2008 and 2010, and that death was identified to be HIV-related using verbal autopsy data.

The target sample was 400 individuals, with power calculations determining it to be adequate for a description of the health and wellbeing of older people in the study area. Having at least 100 people in each group allowed us to test for statistically significant differences between the groups, at 5% level of significance. It was also determined to be appropriate for proposed cross-site analyses under the WHO SAGE programme.

Before data collection, the study questionnaire was translated from English to Zulu and then back-translated by local staff. The questionnaire was tested in a pilot study and revised. The size of the pilot sample was 10% of the target main sample; individuals included in the pilot were not included in the main study. Data were collected by two trained professional nurses. A total of 422 individuals participated in the study, due to the incidence of there being more than one older person in some households, particularly in groups 3 and 4. All persons meeting the inclusion criteria in a visited household were offered the opportunity to participate.

The starting point for selection of participants into groups 1 and 2 was the Hlabisa HIV Treatment and Care Programme [40]. This is a South African Department of Health programme run in partnership with the Africa Centre, from which persons in the Antiretroviral Therapy Evaluation and Monitoring Information System (ARTemis) database were selected to be invited to participate based on inclusion criteria. ARTemis captures information relating to all HIV-infected people accessing HIV care at any one of the 17 primary health care clinics and the district hospital within the Hlabisa sub-district and served as the sampling frame for groups 1 and 2. Around 40% of individuals in the Hlabisa HIV Treatment and Care Programme reside in the Africa Centre surveillance area [40]. With appropriate ethical approval, information collected from the Africa Centre surveillance activities were linked to information collected in the Treatment and Care Programme and those that met the criteria for groups 1 and 2 were randomly selected and approached for informed consent. Group 3 participants were selected by first identifying all adults (18–49 years) in ARTemis who were also under demographic surveillance. Their households were then identified and any person aged 50+ in those households was approached for inclusion in the study. Group 4 participants were selected by identifying all deaths between 2008 and 2010 of adult household members (18–49 years) resident in the surveillance area, and the death was classified as HIV-related using verbal autopsy data. A random sample of older people who were identified to have been co-resident with the adult at the time of death was then drawn, and approached for inclusion. Study instruments are available on request and at http://www.who.int/healthinfo/systems/sage.

### Analytical methods

For analyses in this paper, participants in group 1 and 2 were combined into one ‘HIV-infected’ group because their health status scores were not statistically significantly different from each other. It was hypothesized that HIV-infected people (groups 1 and 2) would have poorer health status than HIV-affected people (groups 3 and 4), since the former are likely to suffer opportunistic infections as a result of HIV which potentially impact upon their physical, mental and emotional well-being. Within the HIV-infected group, considering the pharmacodynamics of ART medications, it was hypothesized that those on ART for three months or less would have poorer health than those on treatment for a year or longer [41].

Chi-square was used to test the significance of the relationship between variables in bivariate analyses. Ordered logistic regressions [42] were used to assess the relationship between factors potentially associated with health. Ordered logistic analysis is an alternative to binary logistic models which avoid arbitrary dichotomisation of an outcome variable that has more than two levels [43]. Ordered logistic regression findings are interpreted as the proportional odds to move from one level of the response variable relative to all other levels of the response variable for a one unit change in the predictor variable [44]. In this analysis, the ordering of the outcome variable was based on quintiles, where the first quintile represented the poorest health and the fifth quintile the best health in each of the three variables described in the next section below. An alpha of 0.05 was set for statistical significance. All analyses were conducted in Stata 11.2 [45].

### Outcome variables: Functional ability (WHODAS), quality of life (WHOQoL) and health state score (HSS)

In this analysis, three measures were used as outcome variables to describe the health and wellbeing of older people: 1) functional ability, 2) quality of life/subjective well-being; and, 3) composite health state score.

In the survey information on health status in eight domains of health (mobility, self-care, affect, vision, pain/discomfort, sleep/energy, interpersonal activities, and cognition) was collected. Functional ability was measured by the 12-item WHO Disability Assessment Schedule, version 2 (WHODAS-II) [46], designed to measure disability from responses to questions on physical functioning in a range of activities of daily life as well as instrumental activities of daily life. Participants were asked about difficulties in the last 30 days with performing activities of daily living such as walking, standing, stooping, kneeling or crouching, getting up from sitting position, getting up from lying down position, picking up things from the table, doing household chores as well as instrumental activities of daily living like getting dressed, bathing, eating, getting to the toilet, using public transport and participation in community activities. Responses to these questions were scored using a five-point likert-type response scale, ‘none’, ‘mild’, ‘moderate’, ‘severe’, and ‘extreme/cannot do’. The computed WHODAS score ranged from 0–36 and was later transformed into 0–100 with 100 being severe/extreme disability. To make the WHODAS measure consistent with the other two measures of health to be employed in this paper, it was inverted (WHODASi) so that a low score indicated low physical functioning ability (high disability) and a high score, high functioning ability (low disability).

The WHO defines quality of life as an “individual’s perception of their position in life in the context of the culture and value systems in which they live and in relation to their goals, expectations, standards and concerns.” [47]. Quality of life or subjective mental well-being was measured using the 8-item WHO Quality of Life (WHOQoL) instrument [47], derived from responses to questions about a participant’s satisfaction with among other things, their self, health, living conditions, personal relationships, ability to perform daily living activities, and their life as a whole. The computed WHOQoL score ranged from 8–40. As with the WHODAS, the WHOQoL score was then transformed into a scale of 0–100; where 100 corresponded to best quality of life.

The composite health state score was derived from questions in the parsimonious set of health domains described above, by applying Rasch models in the Winstep statistical package (http://www.winsteps.com). The underlying theorem in these models is Item Response Theory (IRT), which uses maximum likelihood estimation to combine the pattern of responses to the health domains with the characteristics of each specific item, to arrive at the final health score [48-50]. The health state score combined the questions used to compute the functional ability and the quality of life scores. The health state score was scaled from 0–100 with 100 representing best health. Transformations of the WHODAS, WHOQoL and HSS to be on the same scale eased description and comparisons of the measures, which were then divided into quintiles for further analyses.

### Control variables

The independent factors considered in this analysis, informed by the literature [10], were sex, age group, marital status, household headship, education attainment, income source, household wealth quintiles and rural/urban place of residency. Advancing age is strongly linked to health status. For this analysis, three age groups, 50–59, 60–69 and 70+, were used. Marital status was categorised into never married, currently married and previously married (which included participants reporting to be widowed, divorced or separated). Household headship was used as a proxy for independence and responsibility for care and support of the household. It was categorised into self, spouse or any other person. Education attainment was categorised into: no formal education (including those who only attended adult education classes), completed 6 years or less, or completed more than 6 years of education. The latter two categories agree with UNESCO’s standard classification into primary and secondary level of education [51]. Income source was based on whether a participant had no income, had a government grant (mostly old-age pension grant) or had other income source. Household wealth was measured from possession of assets such as television, radio, and fridge as well as access to amenities like electricity, water, and toilet facilities. Principal component analysis was used to derive household wealth scores which were later categorised into quintiles. Place of residency was divided into rural or urban.

### Ethical clearance

For household and demographic surveillance in the Africa Centre’s Demographic Information System (ACDIS), oral informed consent was obtained from a proxy household respondent, usually the household head but in his/her absence any competent adult household member. For the individual sexual behaviour and HIV surveillance, written informed consent was obtained from each individual participant. In WOPS written informed consent was obtained from all participants; they had to sign or thumb-print the consent form. The Africa Centre Surveillance was approved in 2000 by the ethics committee of the University of KwaZulu-Natal, with annual re-certification since then. For the WOPS, approval for the study was in the first instance obtained from the local community via the community advisory board (CAB) and then the University of KwaZulu-Natal Biomedical Research Ethics Committee (Ref No. BF136/09).

## Results

**Figure 1:**
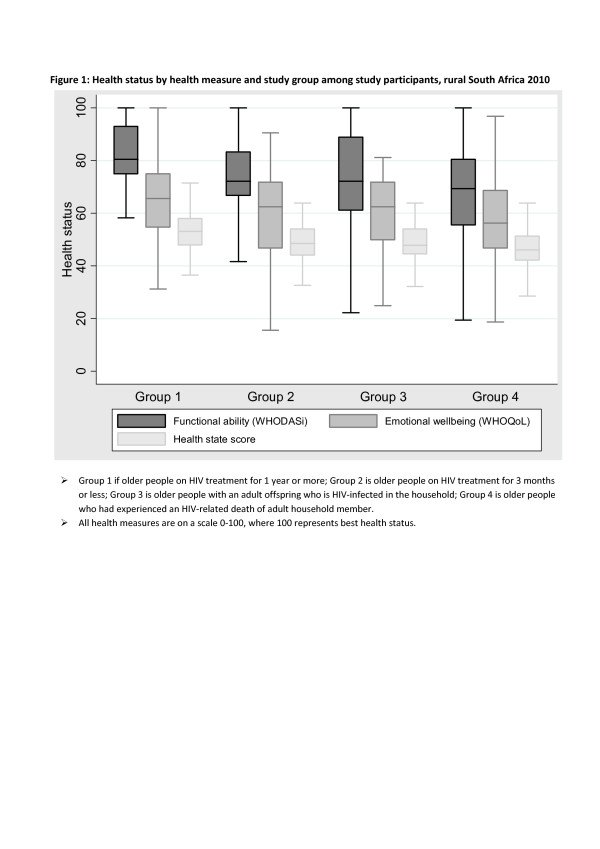
**Health status by health measure and study group among study participants, rural South Africa 2010.** Note ➣ Group 1 is older people on HIV treatment for 1 year or more; Group 2 is older people on HIV treatment for 3 months or less; Group 3 is older people with an adult offspring who is HIV-infected in the household; Group 4 is older people who had experienced an HIV-related death of adult household member. ➣ All health measures are on a scale 0–100, where 100 represents best health status.

In total 316 women and 106 men participated in the study. The median age of the 422 participants was 60 years (range 50–94). Table 1 presents the distribution of participants by socio-demographic characteristics and study group. Significant differences in the study group distributions were observed by age groups, marital status, highest education level attained and source of income (Table 1). Participants in groups 1 and 2 were significantly younger and less likely to be married than those in groups 3 and 4. The scores of the three health measures (WHODAS, WHOQoL and HSS), on a scale of 0–100, where 100 is best health status are presented by study groups in Figure 1. For all four groups, the median scores on the functioning WHODAS measure were higher than for the other two measures of health status. The lowest median scores, with comparatively lower variability, were observed for HSS (Figure 1). Overall comparisons of HIV-infected (groups 1 and 2) to HIV-affected participants (groups 3 and 4) showed significant differences between the two groups with regard to socio-demographic characteristics of age, marital status, education, source of income and place of residency, but not for household headship and household wealth quintiles (results not shown), confirming the need for separate analyses by HIV status.

**Table 1:** Background characteristics by study group of WOPS participants, rural South Africa 2010

	**Group 1**	**Group 2**	**Group 3**	**Group 4**	
Characteristics	n(%)	n(%)	n(%)	n(%)	p-value
**n**	100(23.7)	103(24.4)	107(25.4)	112(26.5)	
**Age group**					<0.001
50–59	60(60.0)	76(73.8)	28(26.2)	26(23.2)	
60–69	34(34.0)	21(20.4)	36(33.6)	37(33.0)	
70–79	6(6.0)	6(5.8)	43(40.2)	49(43.8)	
**Marital status**					0.004
Never married	30(30.0)	42(40.8)	20(18. 7)	24(21.4)	
Married	45(45.0)	45(43.7)	62(57.9)	54(48.2)	
Previously married	25(25.0)	16(15.5)	25(23. 4)	34(30.4)	
**Household Head**					0.265
Self	69(69.0)	56(54.4)	56(52.3)	63(56.3)	
Spouse	14(14.0)	23(22.3)	27(25.2)	23(20.5)	
Other	17(17.0)	24(23.3)	24(22.4)	26(23.2)	
**Education**					0.001
NFE/AEO	32(32.0)	43(41.7)	63(58.9)	63(56.3)	
6 years or less	43(43.0)	36(35.0)	33(30.8)	29(25.9)	
More than 6 years	25(25.0)	24(23.3)	11(10.3)	20(17. 9)	
**Source of income**					0.003
None	8(8.0)	18(17.5)	7(6.5)	7(6.3)	
Other	15(15.0)	18(17.5)	9(8.4)	8(7.1)	
Grants	77(77.0)	67(65.0)	91(85.0)	97(86.6)	
**Wealth quintile**					0.922
First	17(17.0)	27(26.2)	21(19.6)	28(25.0)	
Second	17(17.0)	20(19.4)	20(18.7)	19(17.0)	
Third	23(23.0)	20(19.4)	22(20.6)	21(18.8)	
Fourth	20(20.0)	21(20.4)	24(22.4)	21(18.8)	
Fifth	23(23.0)	15(14.6)	20(18.7)	23(20.5)	
**Place of residency**					0.225
Peri-Urban	48(48.0)	49(47.6)	45(42.1)	40(35.7)	
Rural	52(52.0)	54(52.4)	62(57.9)	72(64.3)	

Note: Group 1 is older people on HIV treatment for 1 year or more; Group 2 is older people on HIV treatment for 3 months or less; Group 3 is older people with adult offspring who are HIV-infected in the household; Group 4 is older people who had experienced an HIV-related death of adult household member.

Table 2 presents the median health scores of HIV-infected and HIV-affected older people by gender and overall. For men, the health scores did not differ significantly between the HIV-infected and the HIV-affected groups. Among women on the other hand, those who were HIV-infected had better functional ability and overall health state than those HIV-affected. For both sexes combined, median health scores were substantially higher for HIV-infected than HIV-affected older people for all three health measures (Table 2). This was evidently driven by women who made up 75% of the sample. Comparing the health scores presented in Table 2 for men who are HIV-infected to women who are HIV-infected, and likewise among the HIV-affected participants, revealed that men reported better health than women in both HIV-infected and HIV-affected participants. Further tests of these (data not presented) showed that there were statistically significant gender differentials in functional ability (p-value < 0.001) and health state score (p-value = 0.002) amongst HIV-infected participants, with men reporting better health than women. Gender differences in functional ability (p-value <0.001) were also found among HIV-affected participants, but not in health state score.

**Table 2:** Median health scores for HIV-infected and HIV-affected older people, rural South Africa 2010

**Measure**	**HIV-infected**	**HIV-affected**	**p-value**
	**Males**		
Functional ability	87.5	81.9	0.551
Quality of life	68.8	64.1	0.550
Health state score	54.1	50.7	0.551
	**Females**		
Functional ability	75.0	69.4	0.002
Quality of life	62.5	59.4	0.276
Health state score	49.2	47.3	0.041
	**Both sexes**		
Functional ability	77.8	69.4	<0.001
Quality of life	62.5	59.4	0.011
Health state score	50.7	47.3	0.001

p-values show test of equality of medians comparing HIV-infected to HIV-affected for males and females separately. A p-value < 0.05 indicates statistically significantly different medians.

### Correlates of health and well-being among study participants

We further examined the association of HIV status with the health and well-being of older people in three separate multivariable models for functional ability, quality of life and composite health state respectively. Each model was adjusted for age, gender, household headship, marital status, education, income source, household wealth quintiles, place of residency and HIV category. Results are presented in Table 3. We found that older people who were HIV-affected via having an adult household member who was HIV positive (group 3) were associated with higher adjusted odds of reporting higher functional ability, quality of life and overall health state, compared to older people who were HIV-affected through a recent HIV-related death of an adult household member (group 4). HIV-infected older people (groups 1 and 2) were similarly significantly associated with higher adjusted odds of being in good physical functioning ability, emotional well-being and health state relative to older people who were HIV affected via HIV-related death of an adult household member between 2008 and 2010. In each of the models presented in Table 3, we tested for interactions between age and gender, and between age and HIV. None of these interaction terms were statistically significant.

**Table 3:** Factors associated with health and well-being adjusted for HIV category, rural South Africa 2010

	**Functional ability**	**Quality of life**	**Health state**
	aOR(95%CI)	aOR(95%CI)	aOR(95%CI)
**HIV category**			
HIV-affected (via HIV-related adult child death)	1.00	1.00	1.00
HIV-affected (with HIV-infected child adult)	2.54(1.02–6.35)	2.52(1.13–5.61)	2.64(1.19–5.88)
HIV-infected	1.91(1.05–3.48)	1.81(1.02–3.19)	1.90(1.09–3.33)
**Sex**			
Male	1.00	1.00	1.00
Female	0.21(0.11–0.39)	0.76(0.41–1.41)	0.30(0.16–0.57)
**Age group**			
50–59	1.00	1.00	1.00
60–69	0.79(0.38–1.65)	1.76(0.85–3.64)	1.01(0.49–2.10)
70+	0.19(0.10–0.37)	0.49(0.26–0.93)	0.24(0.13–0.47)
**Marital Status**			
Never Married	1.00	1.00	1.00
Married	0.83(0.46–1.49)	2.17(1.20–3.94)	1.26(0.74–2.17)
Previously Married	3.33(1.57–7.05)	0.67(0.34–1.35)	1.82(0.94–3.51)
**Household Head**			
Self	1.00	1.00	1.00
Spouse	1.21(0.60–2.44)	0.74(0.39–1.39)	1.05(0.53–2.08)
Other	0.95(0.54–1.70)	1.13(0.60–2.11)	1.05(0.61–1.78)
**Education level**			
NFE/AEO	1.00	1.00	1.00
6 years or less	1.47(0.88–2.45)	1.40(0.85–2.33)	1.46(0.86–2.45)
More than 6 years	3.38(1.72–6.65)	1.37(0.75–2.51)	2.49(1.37–4.55)
**Source of income**			
None	1.00	1.00	1.00
Other	1.33(0.44–3.99)	3.70(1.22–11.19)	2.34(1.01–5.43)
Grants	1.17(0.42–3.23)	2.33(0.93–5.79)	1.75(0.80–3.80)
**Wealth quintiles**			
First	1.00	1.00	1.00
Second	0.61(0.28–1.36)	1.65(0.84–3.23)	0.94(0.45–1.99)
Third	0.71(0.35–1.44)	1.65(0.79–3.42)	0.84(0.42–1.69)
Fourth	1.22(0.56–2.66)	2.56(1.24–5.31)	1.55(0.74–3.25)
Fifth	0.55(0.25–1.22)	2.28(1.08–4.78)	0.88(0.44–1.77)
**Rural/urban**			
Urban	1.00	1.00	1.00
Rural	3.00(1.06–8.47)	1.88(0.59–5.94)	2.51(0.74–8.50)

aOR = Adjusted Odds Ratios; HIV category variable adjusted for all covariates listed in the Table. Each socio-economic variable adjusted for HIV plus the remaining covariates listed in the Table.

NFE/AEO = No formal education or adult education only.

Previously married = Separated, divorced and widowed participants.

In stratified analyses of HIV-infected and HIV-affected older people, similar factors were associated with WHODAS (Table 4) and HSS (Table 5). Gender was strongly linked to WHODAS and HSS measures among study participants. Adjusting for age, marital status, household headship, education level, household socio-economic status, source of income and place of residency, older women were 70-80% less likely to report being in good functional ability and overall health state than older men. The oldest age category in both HIV-infected and HIV-affected older people was statistically significantly associated with lower odds of being in good functional ability and health state in the adjusted models. Among HIV-affected older people, being previously married and having more than six years of education were significantly associated with higher adjusted odds of better functional ability and health state. Adjusting for other factors in the model, HIV-infected older people who had some source of income compared to none were significantly more likely to report a high health state score (Table 5).

**Table 4:** Factors associated with functional ability (WHODAS) stratified by HIV category, rural South Africa 2010

	**HIV-Infected**		**HIV-Affected**	
	Unadjusted	Adjusted	Unadjusted	Adjusted
	OR [95% CI]	aOR [95% CI]	OR [95% CI]	aOR [95% CI]
**Sex**				
Male	1.00	1.00	1.00	1.00
Female	0.25 [0.13–0.48]	0.15 [0.08–0.29]	0.30 [0.12–0.75]	0.20 [0.08–0.50]
**Age group**				
50–59	1.00	1.00	1.00	1.00
60–69	0.81 [0.45–1.47]	0.84 [0.46–1.54]	0.75 [0.37–1.50]	0.46 [0.19–1.13]
70+	0.43 [0.11–1.60]	0.19 [0.05–0.75]	0.34 [0.17–0.67]	0.17 [0.06–0.46]
**Marital Status**				
Never married	1.00	1.00	1.00	1.00
Married	0.92 [0.51–1.66]	0.67 [0.33–1.36]	0.91 [0.50–1.67]	1.08 [0.42–2.74]
Previously married	1.74 [0.80–3.81]	1.80 [0.70–4.63]	2.04 [0.95–4.40]	4.80 [1.61–14.32]
**Household Head**				
Self	1.00	1.00	1.00	1.00
Spouse	0.89 [0.46–1.73]	1.98 [0.87–4.48]	0.82 [0.42–1.60]	1.19 [0.42–3.38]
Other	1.01 [0.50–2.04]	1.21 [0.57–2.53]	0.78 [0.40–1.52]	0.92 [0.44–1.95]
**Education level**				
NFE/AEO	1.00	1.00	1.00	1.00
6 years or less	1.89 [1.02–3.50]	1.90 [1.00–3.61]	1.06 [0.58–1.93]	1.26 [0.63–2.54]
More than 6 years	1.67 [0.85–3.31]	1.70 [0.81–3.54]	3.38 [1.35–8.50]	5.54 [2.00–15.33]
**Source of income**				
None	1.00	1.00	1.00	1.00
Other	1.26 [0.42–3.78]	1.39 [0.43–4.54]	2.27 [0.66–7.86]	1.55 [0.21–11.56]
Grants	1.15 [0.41–3.21]	1.80 [0.68–4.76]	0.87 [0.30–2.55]	1.08 [0.17–7.02]
**Wealth quintiles**				
First	1.00	1.00	1.00	1.00
Second	1.08 [0.42–2.82]	1.73 [0.66–4.52]	0.38 [0.15–0.94]	0.44 [0.14–1.39]
Third	1.46 [0.65–3.27]	1.81 [0.72–4.57]	0.55 [0.23–1.33]	0.52 [0.19–1.42]
Fourth	1.33 [0.59–2.98]	1.48 [0.64–3.45]	1.39 [0.52–3.69]	1.24 [0.40–3.78]
Fifth	1.02 [0.43–2.42]	1.40 [0.49–4.03]	0.49 [0.21–1.11]	0.38 [0.12–1.15]
**Rural/urban**				
Urban	1.00	1.00	1.00	1.00
Rural	0.82 [0.42–1.60]	1.03 [0.46–2.30]	0.79 [0.28–2.27]	1.30 [0.48–3.56]

aOR = Adjusted Odds Ratios; Adjusted for all covariates listed in the Table.

NFE/AEO = No formal education or adult education only.

Previously married = Separated, divorced and widowed participants.

**Table 5:** Factors associated with health state (HSS) stratified by HIV category, rural South Africa 2010

	**HIV-Infected**		**HIV-Affected**	
	Unadjusted	Adjusted	Unadjusted	Adjusted
	OR [95% CI]	aOR [95% CI]	OR [95% CI]	aOR [95% CI]
**Sex**				
Male	1.00	1.00	1.00	1.00
Female	0.30 [0.17–0.54]	0.20 [0.11–0.37]	0.37 [0.15–0.95]	0.31 [0.12–0.81]
**Age group**				
50–59	1.00	1.00	1.00	1.00
60–69	0.93 [0.51–1.68]	0.94 [0.45–1.95]	0.92 [0.46–1.86]	0.70 [0.30–1.64]
70+	0.51 [0.15–1.76]	0.20 [0.05–0.73]	0.35 [0.18–0.67]	0.25 [0.10–0.58]
**Marital Status**				
Never married	1.00	1.00	1.00	1.00
Married	1.22 [0.67–2.21]	0.96 [0.44–2.09]	1.41 [0.77–2.57]	1.53 [0.69–3.39]
Previously married	0.95 [0.42–2.15]	0.86 [0.36–2.06]	1.49 [0.68–3.24]	2.79 [1.11–7.02]
**Household Head**				
Self	1.00	1.00	1.00	1.00
Spouse	0.70 [0.37–1.32]	0.95 [0.44–2.06]	1.16 [0.56–2.39]	1.30 [0.48–3.54]
Other	0.79 [0.41–1.52]	0.91[0.43–1.94]	0.89 [0.48–1.64]	1.31 [0.63–2.75]
**Education level**				
NFE/AEO	1.00	1.00	1.00	1.00
6 years or less	1.65 [0.89–3.05]	1.72 [0.87–3.39]	1.38 [0.73–2.64]	1.36 [0.68–2.74]
More than 6 years	1.71 [0.88–3.33]	1.29 [0.63–2.64]	3.76 [1.64–8.63]	4.50 [1.76–11.51]
**Source of income**				
None	1.00	1.00	1.00	1.00
Other	2.67 [1.04–6.85]	3.67 [1.15–11.66]	2.59 [0.94–7.12]	1.72 [0.36–8.13]
Grants	1.72 [0.85–3.48]	3.13 [1.34–7.28]	1.14 [0.46–2.83]	1.31 [0.32–5.34]
**Wealth quintiles**				
First	1.00	1.00	1.00	1.00
Second	1.68 [0.69–4.09]	2.17 [0.85–5.52]	0.73 [0.31–1.71]	0.78 [0.28–2.18]
Third	1.69 [0.78–3.66]	1.86 [0.77–4.52]	0.87 [0.36–2.09]	0.70 [0.26–1.90]
Fourth	2.23 [1.04–4.76]	2.52 [1.09–5.84]	1.77 [0.65–4.84]	1.49 [0.52–4.23]
Fifth	2.27 [1.00–5.17]	2.65 [0.85–8.19]	0.86 [0.38–1.95]	0.59 [0.22–1.56]
**Rural/urban**				
Urban	1.00	1.00	1.00	1.00
Rural	1.26 [0.66–2.39]	1.41 [0.66–2.99]	1.28 [0.51–3.23]	2.05 [0.78–5.36]

aOR = Adjusted Odds Ratios; Adjusted for all covariates listed in the Table.

NFE/AEO = No formal education or adult education only.

Previously married = Separated, divorced and widowed participants.

Table 6 presents the quality of life results. For quality of life or subjective well-being, having some source of income and being in the upper two wealth quintiles were highly associated with the likelihood of having a high quality of life among HIV-infected older people even after adjusting for other factors in the model. Being female and having been previously married were associated with lower odds of good quality of life in HIV-infected older people, in both univariate and multi-variate models. Amongst HIV-affected participants, being currently married was associated with better quality of life even after adjustments. We also found that having some level of education compared to none was associated with higher likelihood of better quality of life in HIV-affected older people, although statistical significance was not reached after adjusting for other variables. Among HIV-affected older people, being in the fourth household wealth quintile and residing in the rural segment of the surveillance area were other factors strongly associated with better quality of life, in unadjusted and adjusted analyses.

**Table 6:** Factors associated with Quality of Life (WHOQoL) stratified by HIV category, rural South Africa 2010

	**HIV-Infected**		**HIV-Affected**	
	Unadjusted	Adjusted	Unadjusted	Adjusted
	OR [95% CI]	aOR [95% CI]	OR [95% CI]	aOR [95% CI]
**Sex**				
Male	1.00	1.00	1.00	1.00
Female	0.53 [0.29–0.96]	0.40 [0.21–0.79]	0.75 [0.35–1.60]	0.88 [0.34–2.28]
**Age group**				
50–59	1.00	1.00	1.00	1.00
60–69	1.20 [0.64–2.26]	1.47 [0.67–3.25]	1.06 [0.53–2.09]	0.96 [0.45–2.04]
70+	0.76 [0.24–2.43]	0.26 [0.07–1.01]	0.63 [0.31–1.25]	0.62 [0.26–1.50]
**Marital Status**				
Never married	1.00	1.00	1.00	1.00
Married	1.66 [0.91–3.03]	1.73 [0.80–3.73]	2.54 [1.22–5.28]	2.39 [1.02–5.61]
Previously married	0.39 [0.17–0.92]	0.32 [0.13–0.80]	0.85 [0.34–2.16]	0.97 [0.37–2.57]
**Household Head**				
Self	1.00	1.00	1.00	1.00
Spouse	0.89 [0.46–1.70]	0.70 [0.33–1.49]	1.21 [0.64–2.29]	0.86 [0.34–2.16]
Other	0.94 [0.45–1.99]	0.98 [0.40–2.45]	0.78 [0.38–1.62]	1.28 [0.53–3.11]
**Education level**				
NFE/AEO	1.00	1.00	1.00	1.00
6 years or less	1.20 [0.64–2.25]	1.15 [0.59–2.25]	2.29 [1.23–4.25]	1.59 [0.81–3.12]
More than 6 years	1.31 [0.67–2.56]	0.69 [0.33–1.46]	2.71 [1.17–6.27]	2.24 [0.91–5.52]
**Source of income**				
None	1.00	1.00	1.00	1.00
Other	4.92 [1.84–3.15]	6.81 [2.04–2.73]	3.07 [0.60–15.61]	2.25 [0.33–15.13]
Grants	2.77 [1.41–5.46]	3.89 [1.56–9.70]	1.78 [0.53–6.00]	1.68 [0.31–9.03]
**Wealth quintiles**				
First	1.00	1.00	1.00	1.00
Second	2.11 [0.93–4.75]	2.42 [1.02–5.76]	1.97 [0.83–4.68]	1.71 [0.68–4.33]
Third	1.83 [0.77–4.39]	2.40 [0.91–6.30]	2.39 [0.89–6.42]	1.91 [0.69–5.28]
Fourth	2.84 [1.33–6.09]	3.70 [1.45–9.45]	3.20 [1.21–8.42]	3.07 [1.04–9.11]
Fifth	3.38 [1.62–7.08]	3.85 [1.36–10.92]	3.13 [1.22–8.07]	2.20 [0.83–5.80]
**Rural/urban**				
Urban	1.00	1.00	1.00	1.00
Rural	1.72 [0.76–3.91]	1.25 [0.50–3.10]	2.17 [1.31–3.60]	2.93 [1.44–5.98]

aOR = Adjusted Odds Ratios; Adjusted for all covariates listed in the Table.

NFE/AEO = No formal education or adult education only.

Previously married = Separated, divorced and widowed participants.

## Discussion

Our results indicate that the majority of older people aged 50-plus years rated their functional ability favourably (median by gender and HIV status from 69 to 88). The lowest scores were observed for the multi-dimensional health state, suggesting a much lower health status than reported using the functional ability or quality of life measures separately. These differences could partly be explained by the underlying methodology in computing the scores for these health measures (WHO tools used for functional ability and quality of life assessment are simple arithmetic additive scores, while the health state score was generated using Item Response Theory and Rasch models). However, in spite of these differences in methodologies, correlates of the WHODAS and HSS scores were very similar for both HIV-infected and HIV-affected people. This may be reflective of the stronger contributions from the health domains describing physical functioning than those that are more subjective to the composite health state score. Our findings highlight and support previous findings that the use of a single health outcome measure may be helpful to describe the overall health status of older people, but may also have limitations [52]. Using only the composite health score would have underestimated the health status of our study participants. An investigation of the contributions of the specific domains to overall health status needs to be undertaken for a more precise description of the health of older people and to inform the design of interventions [53].

Our findings suggest that the effect of being HIV-affected differs between those who are affected via having an HIV-infected adult child and those affected via an HIV-related death of an adult child. Older people who had lost an adult child due to HIV were more likely to be in poor physical and emotional health than those with a living HIV-infected adult child (Table 3). The death of an adult child is likely to take its toll on the physical health of older people who have had to care for the adult during the time of sickness [54,55] and who may additionally be emotionally affected upon death [31]. The death of an adult child may furthermore place greater household responsibilities on the older person as there may be loss of household income from the deceased adult [29,30,55] as well as orphaned grandchildren who may require financial and social support. Thus death of an adult child is likely to strain the older person physically, emotionally and financially, which in turn is likely to contribute to their poorer physical and emotional health relative to older people still living with an infected adult.

Overall, combining the two HIV-affected groups into one, we further found that HIV-infected participants had better functional ability, quality of life and overall health state than HIV-affected participants. This may seem counterintuitive in that ill-health may be expected to be more prevalent among HIV-infected people [41,53], but this difference may partly be explained by the enhanced health care that this group receives as part of their regular clinic visits for antiretroviral (ART) treatment. These findings are consistent with a study by Louwagie et. al. [16] who compared health related quality of life (QoL) of patients on Highly Active Antiretroviral Treatment (HAART) to those awaiting HAART, and showed that patients on HAART had on average a higher health-related QoL score than those awaiting HAART. Other studies in South Africa have also demonstrated the beneficial effects of HIV treatment on health and well-being. In a study in the Free-State province of South Africa, where changes over a 12-month period in the physical and emotional quality of life of people on ART were examined [17], it was shown that at follow-up people on treatment had fewer adverse events than at baseline; adverse events were negatively associated with physical and emotional quality of life. Evidence of ART leading to improved health can also be inferred from previous work in our study community that showed that ART has contributed to declining mortality among adults [56]. The considerable evidence that HIV treatment is effective in achieving sustained improvement in the health and well-being of HIV-infected people [17,57], clearly contributes to the superior health status of HIV-infected people we observe.

Our results do suggest that as age advances irrespective of HIV status, our study population in rural South Africa is increasingly associated with poorer functional ability and overall health state, with major gender differences. Women reported poorer health status than men among both HIV-infected and HIV-affected participants. These results showing a male advantage in self-reported health in later life are consistent with other studies [11,58-62]. A pooled analysis of data collected in four African and four Asian sites, whose study instruments as in the present study were adapted from WHO’s Study on global AGEing and adult health (SAGE), reported that older women had significantly lower health scores than older men at all age groups [10]. According to findings from a nationally representative study from Thailand [63], a larger part of women’s remaining life expectancy in old-age is spent in a disabled state. These gender differentials in health are said to be more complicated and nuanced than can be explained by biological or medical factors alone [64]. Hirve and colleagues [65] argue that this female disadvantage in health may be accounted for by advancing age, societal norms concerning women, occupation, lower education attainment and lower empowerment. The societal norms and institutionalisation that tend to fuel this sex disparity in health mostly occur around life’s central foci of ‘paid work or unpaid family work’ [64].

In South Africa, people in the age range considered for this study come from a generation renowned for migration of men to the mines and cities for paid work while the women remained in their rural homes with the care burden for children and those with disabilities [66]. This has meant that men and women are exposed to different health-related risks as well as resources across the life-course, and has highly likely contributed to the sex disparities in health we observe. Independent of HIV status, older women are clearly more vulnerable than men to poorer health and functional ability limitations, which are a function of circumstances over the life course.

Being in the highest two household wealth quintiles was strongly related with better quality of life, even after adjusting for other factors in HIV-infected participants. This is consistent with a study among older people aged 50+ in Pune district, India, which found that older people in higher household wealth quintiles were more likely to report better quality of life than those in lower wealth quintiles [65]. However, our results and context differ from the Pune district study in that in their case there was no ready access to government cash transfers and they did not find a significant association between gender and quality of life. In our study area government cash transfers in the form of old-age pensions are widely available and we find a significant association between gender and quality of life, as well as between having some income source and quality of life. Most of this income, which is linked to the quality of life of older people in rural South Africa, is from non-contributory government cash transfers or grants; therefore rapid increases in the proportion of older people poses serious challenges to their well-being by threatening the sustainability of the cash transfers programme.

Gender, advancing age, education and income were independently strongly associated with the health and well-being of older people in this study. The factors reported here associated with health status were similarly reported on by others using similar study instruments [10,11,53,60,61,65,67-69]. In six of these studies all individuals aged 50+ were eligible for inclusion and in two studies a random sample of households containing at least one older person aged 50+ was targeted. For this study, individuals among HIV-infected and HIV-affected clusters of older people within the community were selected. Another main methodological difference to these other studies is that they applied binary logistic regressions to the quintile health scores, where they defined those in the highest two quintiles as healthy and the rest as unhealthy. The decision as to at which quintile the cut-off into healthy and unhealthy should be is highly arbitrary [43,44] and different results may be obtained if different cut-off points are used. Ordered logistic regression analyses, which make use of the quintile distributions without an arbitrary cut-off, were used in this study. Despite these methodological differences, the findings confirm that health and wellbeing of older people varies by socio-demographic characteristics such as age group, gender, education attainment and income, but is further strongly linked to whether the older person is HIV-infected or HIV-affected.

There are, however, some limitations to our findings. In addition to our small sample sizes, participants into our study were purposefully sought to be categorised into HIV-infected or HIV-affected groups - this could have biased our findings. Some of the potential sampling bias was corrected by applying sampling weights and making use of survey tools in the statistical software package used in the analyses. Another limitation of the analysis is the possibility for some of the participants categorised as HIV-affected to also be HIV-infected themselves. This is likely to occur if such participants were tested and/or accessing HIV treatment outside of the Hlabisa sub-district or from private practitioners and hence not captured in the Hlabisa HIV Treatment and Care Programme. It is, however, highly unlikely that participants were accessing treatment and care outside the sub-district given the logistical and financial implications of travelling significant distances particularly for older people. Thus, this potential bias was assumed to be very negligible. Although a blood specimen was collected from all participants as per study protocol these specimens were not tested for HIV antibodies. All participants were informed that no HIV testing would be done on the specimens. However, an earlier study from our study population showed that HIV prevalence in the population 50+ in 2008 was 9.5% (95% CI 8.4-10.7), with an incidence rate of 0.5% (95% CI 0.3–1.0) [35]. Therefore, we do not expect to have had many infected people in groups 3 or 4 to significantly bias our findings.

We urge caution in the interpretation of our results, particularly the association of age with poorer functional ability and health state because of small-numbers, especially in the oldest age group, and limited statistical power. The results may also have been affected by a healthy selection effect into the WOPS - those that participated in the study may be survivors from their cohorts. Furthermore, the study was cross-sectional, thus it is not possible to make causal inferences between the socio-demographic factors considered and health status. We are limited in generalising our findings to the general older population of South Africa since our study participants came from a population under constant surveillance with ready access to a comprehensive HIV care and treatment programme [40]. In addition we have not controlled for other household factors such as number of HIV-infected persons in the household, living arrangements (living alone, in skip-generation household or multigenerational household) and cash transfers to other household members as that was beyond the scope of this analysis. Our results nonetheless make an important contribution to understanding the correlates of health and well-being of older people in rural South Africa.

## Conclusions

Both HIV-infected and HIV-affected women reported poorer health status than men. Addressing the poorer health status in older women will require a life-course perspective targeting the varied contributory factors to women’s disadvantage in health in later life. Some of the factors contributing to women’s poorer health in later life include limited access to education, the labour market and means of production such as land over the life course. There is need for policy interventions and a change of societal norms regarding paid work and unpaid family work to ensure women are not overburdened with care responsibilities, which contributes to their poorer health status in later life.

There is urgent need for the healthcare system in South Africa to start responding to the needs of the increasingly ageing population. Over the past decade considerable resources have been dedicated to HIV care and management in terms of manpower, infrastructure, and interventions, which has greatly contributed to improving the overall health and well-being of HIV-infected older people. The same level of resources have not been devoted to the general health and well-being of all older people, with the care and management of chronic health conditions associated with advancing age like hypertension, diabetes, cancer and cardiovascular diseases receiving less attention than the health burden in other age groups. As such older people who are not HIV-infected may be at increased vulnerability of poorer health than those who are HIV-infected. Policy and programme interventions are urgently needed given increasing numbers of older people. This study is the first report that HIV-infected older people have better functional ability and health state than HIV-affected older people - a finding that necessitates further research especially using population-based data.

## Competing interests

The authors declare that they have no competing interests.

## Authors’ contributions

SC, PK, MN, PM and MLN conceived and participated in the design of the WOPS study. MN participated in the study design, data collection, co-ordination, performed the statistical analysis and drafted the manuscript. JF, ME, PK, VH and MLN gave constructive comments during the analysis and writing of the paper. All authors substantially contributed, read and approved the final manuscript.

## Pre-publication history

The pre-publication history for this paper can be accessed here:

http://www.biomedcentral.com/1471-2458/12/259/prepub
